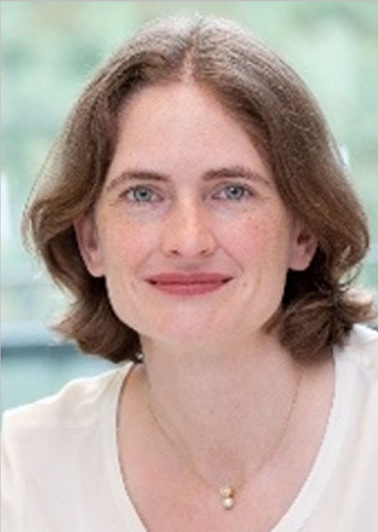# Contemporary Approaches for Site‐Selective Dual Functionalization of Proteins

**DOI:** 10.1002/anie.202012034

**Published:** 2021-02-26

**Authors:** Lujuan Xu, Seah Ling Kuan, Tanja Weil

**Affiliations:** ^1^ Max Planck Institute for Polymer Research Ackermannweg 10 55128 Mainz Germany; ^2^ Institute of Inorganic Chemistry I Ulm University Albert-Einstein-Allee 11 89081 Ulm Germany

**Keywords:** biomedical applications, protein conjugates, protein dual functionalization, site-selective protein modification

## Abstract

Site‐selective protein functionalization serves as an invaluable tool for investigating protein structures and functions in complicated cellular environments and accomplishing semi‐synthetic protein conjugates such as traceable therapeutics with improved features. Dual functionalization of proteins allows the incorporation of two different types of functionalities at distinct location(s), which greatly expands the features of native proteins. The attachment and crosstalk of a fluorescence donor and an acceptor dye provides fundamental insights into the folding and structural changes of proteins upon ligand binding in their native cellular environments. Moreover, the combination of drug molecules with different modes of action, imaging agents or stabilizing polymers provides new avenues to design precision protein therapeutics in a reproducible and well‐characterizable fashion. This review aims to give a timely overview of the recent advancements and a future perspective of this relatively new research area. First, the chemical toolbox for dual functionalization of proteins is discussed and compared. The strengths and limitations of each strategy are summarized in order to enable readers to select the most appropriate method for their envisaged applications. Thereafter, representative applications of these dual‐modified protein bioconjugates benefiting from the synergistic/additive properties of the two synthetic moieties are highlighted.

## Graphical abstract

Site‐selective dual protein functionalization serves as an invaluable tool for investigating protein structures and functions in complicated cellular environments and accomplishing semi‐synthetic protein conjugates. The methodologies, as well as the applications of dual‐functionalized protein bioconjugates are highlighted in this review.

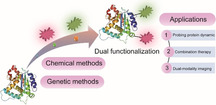

## 1 Introduction

Proteins are ubiquitous in Nature, serving as the basic building blocks of life. They play many essential roles in a myriad of biological processes, such as molecular transport, energy conversion, inter‐ and intramolecular signaling.[^1^, ^2^] Nature expands protein diversity by post‐translational modifications (PTMs) after their biosynthesis in the ribosome, thus vastly enlarging their structural and functional repertoire by up to two orders of magnitude.[^3^, ^4^] Inspired by Nature's elegance, scientists strived to modify proteins with diverse synthetic moieties, allowing for the creation of bioconjugates with high degree of structural perfection and new functional characteristics.^[5]^

In this context, the past decades revealed significant progress in the development of new methodologies for site‐selective protein functionalization to install the desired functionalities at pre‐defined sites.[^3^, ^6^, ^7^, ^8^, ^9^, ^10^] These well‐defined protein conjugates offer great prospects for a wide range of fields including biomedicine, bioimaging, biosensing and materials science.[^5^, ^11^, ^12^, ^13^, ^14^, ^15^, ^16^, ^17^] Some promising examples include the conjugation of synthetic polymers to therapeutic proteins to improve their solubility and extend their plasma circulation half‐life^[13]^ or the attachment of anticancer drugs to antibodies forming antibody‐drug conjugates (ADCs) for cell‐targeted cancer therapy.[^11^, ^12^]

Nevertheless, along with the increasing demand for multifunctional bioconjugates to perform more sophisticated biological studies in vitro as well as in vivo, appending only one type of functionality to proteins is often insufficient to customize proteins for the desired applications. For example, despite the clinical success of ADCs, classical ADCs equipped with a single type of anticancer drug could still suffer from low efficacy, drug resistance, unfavorable pharmacokinetics, immunogenicity, and the inherent hydrophobicity of the drug, which greatly hampers their further in vivo applications.[^18^, ^19^] In addition, there is also an increasing demand for new therapeutic strategies which combine imaging agents and drug molecules within the protein, for example, antibody, for real‐time monitoring during the treatment.^[20]^ In another example, a protein was functionalized with three copies of cell‐targeting somatostatin peptide and an enzyme. Remarkably, the resultant bioconjugate inhibited tumor growth already at 100‐fold lower concentration than a clinically approved antibody acting via a similar mode of action. Furthermore, co‐administration of this protein bioconjugate with an approved anticancer drug, doxorubicin, boosted its antitumor activity in a combination therapy approach.^[21]^ However, these multifunctional protein conjugates have been mainly achieved by statistical modification on the protein surface,[^22^, ^23^] which results in heterogeneous mixtures with reduced protein activity as well as batch‐to‐batch variations. In view of biosafety, these limitations significantly hamper their further developments. Consequently, there is a pressing need to devise new strategies to generate protein bioconjugates that exhibit higher order of structural and functional complexity but retaining structural perfection. In this regard, site‐selective dual functionalization of proteins has emerged as a promising strategy to customize proteins for the respective applications.

**Figure 1:**
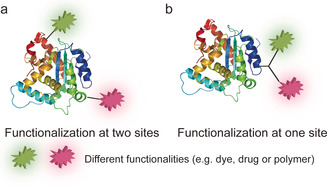
Two different approaches for dual functionalization of proteins (a) at two different sites and (b) at one single site with a multifunctional linker.

In this review, dual functionalization is defined as the incorporation of two different functionalities into proteins in a site‐selective fashion, which is accomplished either at two different amino acids (AA) sites or at a single AA residue at the protein surface (Figure 1). In both cases, the reagents as well as the sequence of the bioconjugation reactions need to be carefully considered. Dual‐modified protein bioconjugates can harness the function and properties imparted by the respective payloads, which complement the capabilities of biomolecules and significantly expand their functional arsenal.^[24]^ For example, dual functionalization with two chromophores at distinct sites allows real‐time monitoring of changes in protein conformations and dynamics upon ligand binding in native environments or in response to certain stimuli by Förster Resonance Energy Transfer (FRET) measurements.^[25]^ This cannot be achieved by mono‐functionalization of proteins. Moreover, a new generation of protein therapeutics/diagnostics can be derived by incorporating two different functionalities, as exemplified by integrating a drug and an imaging agent into one platform for simultaneous therapy and diagnostics applications (theranostics) or inserting two different drugs into an antibody for combination therapy.[^20^, ^26^, ^27^]

**Figure 2:**
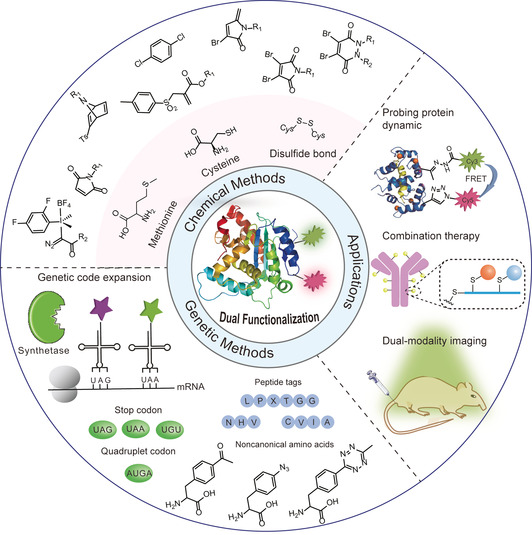
Overview of dual functionalization methodologies for proteins and biomedical applications of the resultant protein conjugates (protein dual functionalization at two different amino acid residues is selected as an example).

Owing to the emerging interest for addressing previously “unanswered” fundamental scientific questions as well as offering practical solutions for biomedical applications, site‐selective dual functionalization of proteins has seen rapid developments in the past ten years. In this review, we first summarize the synthetic strategies for protein dual functionalization to provide a timely overview of this burgeoning field, which serves as a guideline for the selection of the most appropriate method for the envisaged applications (Figure 2). In addition, the rationale and principles behind these synthetic strategies as well as the strength and inherent limitations are discussed. In the last section, some representative applications of dual‐functionalized protein conjugates benefitting from the additive or even synergistic features are highlighted.

## 2 Chemical toolbox for site‐selective functionalization of proteins

Site‐selective protein functionalization either relies on the functionalization of canonical AAs on the protein surface or genetically encoded noncanonical AA (ncAA) bearing the appropriate bioorthogonal groups.^[28]^ Different methodologies have been reported to achieve site‐selective mono‐functionalization of proteins with high efficiency in a residue specific manner. Bioconjugations have been accomplished at the N‐terminus, tyrosine, cysteine or serine residues, to list just a few examples. A brief summary of the commonly used chemical methods for mono‐functionalization of natural AA residues is given in Table 1.[^3^, ^6^, ^7^, ^29^, ^30^, ^31^, ^32^] For more detailed discussions on protein mono‐functionalization, we refer the readers to other excellent reviews on this topic.[^3^, ^6^, ^7^, ^29^, ^30^, ^31^] Among the 20 AAs, unpaired cysteines have become the primary choice for functionalization owing to their low abundance at the protein surface as well as the unique nucleophilicity and versatile reactivity profile of the thiol groups.[^7^, ^33^, ^34^, ^35^] Besides the unpaired cysteine residues, targeting other side chain residues, such as disulfide bonds and methionine, have also emerged as valuable alternatives to the more commonly used cysteine‐based strategies for protein functionalization (Table 1). All these available methods potentially offer a versatile bioconjugation toolbox to achieve dual functionalization of proteins at two different AA sites. For instance, cysteine and methionine residues are exploited for dual modification with maleimide and hypervalent iodine reagents that proceed in a sequential manner without cross reactivity.^[59]^ Despite the simplicity and convenience to achieve dual modification at two different AA residues, there are still only few reports that address two different AA residues at the protein surface. Furthermore, judicious selection and combination of the two mono‐functionalization methods is crucial as the bioconjugation reactions need to be orthogonal and also compatible with each other, to ensure high modification efficiency. Therefore, although other methods involving serine, tyrosine or tryptophan potentially offer a versatile bioconjugation toolbox that is in principle suitable for protein dual functionalization at two different AA residues, these reactions have not been reported yet in this context.

**Table 1:** Brief summary of the currently available chemical toolbox for site‐selective protein functionalization.

AA residues	Methods
Cysteine	Halocarbonyl,^[9]^ Maleimide derivatives,[^33^, ^36^] Sulfone derivatives,[^37^, ^38^] Metal‐mediated arylation[^39^, ^40^, ^41^] Thiol–ene/yne reactions,[^42^, ^43^] Dichlorotetrazine^[44]^ Vinylphosphonothiolate,^[45]^ Ethnylbenziodoxolones^[46]^
	
Disulfide	Allyl sulfone,^[38]^ Dibromomaleimide,^[47]^ Oxetane,^[48]^ Divinylpyridine,^[49]^ Dibromopyridazinediones^[50]^
	
N‐terminus	2‐Formylphenylboronic derivatives,[^51^, ^52^] 2‐Pyridinecarboxyaldehyde,^[53]^ 2‐Ethynylbenzaldehydes,^[54]^ o‐Aminophenols,^[55]^ 2‐Cyanobenzothiazole,^[56]^ Pictet–Spengler reaction,^[57]^ Ketenes^[58]^
	
Methionine	Hypervalent iodine reagent,^[59]^ Oxaziridines,^[60]^ Epoxide^[61]^
	
Tyrosine	Diazonium,^[62]^ Mannich‐type reaction,^[63]^ Pd‐mediated alkylation,^[64]^ Trizoline‐diones^[65]^
	
Tryptophan	Rhodium‐carbenoid,^[66]^ Keto‐ABNO,^[67]^ Metal‐mediated arylation/alkynylation[^68^, ^69^, ^70^, ^71^]
	
Serine	Salicylaldehyde ester^[72]^ Phosphorus–sulfur incorporation reagents^[73]^
	
Arginine	Glyoxal reagents^[74]^

Alternatively, the attachment of a multifunctional linker containing two reactive orthogonal groups to a single site on the protein surface, usually an exposed cysteine or a disulfide bond, also provides straightforward access for the incorporation of two different functionalities. These bioconjugation reagents include maleimide derivatives,^[47]^ sulfone derivatives[^37^, ^38^] and other novel reagents,[^44^, ^46^] which are discussed in Section 3.

**Figure 3:**
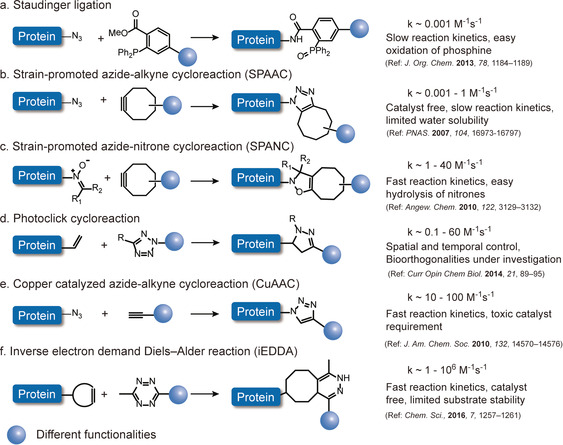
Commonly used bioorthogonal reactions for protein modification and summary of some selected features.

**Figure 4:**
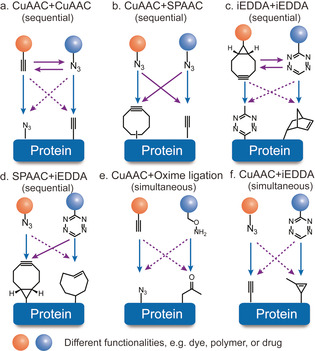
Bioorthogonal chemistry combinations for dual functionalization of proteins (a) CuAAC + CuAAC.^[79]^ (b) CuAAC + SPAAC.^[50]^ (c) CuAAC + iEDDA.^[25]^ (d) SPAAC + iEDDA.^[80]^ (e) CuAAC + Oxime ligation.^[81]^ (f) CuAAC+ iEDDA.^[82]^ (Solid line refers to two moieties reacting with each other; dashed line refers to their orthogonal reactivities).

The recent advances in bioorthogonal chemistry have greatly promoted the progress of site‐selective protein modification. Nowadays, various bioorthogonal reactions have been reported with optimized reaction parameters, such as reaction rate, catalyst type, and substrate stability, to impart the desired functionalities for the envisaged applications.[^75^, ^76^] Some commonly used bioorthogonal chemistries as well as selected characteristics are summarized in Figure 3. The copper‐catalyzed azide‐alkyne cycloaddition (CuAAC) bears the advantage of fast reaction kinetics but suffers from the usage of toxic copper catalyst.^[77]^ Strain‐promoted azide‐alkyne cycloaddition (SPAAC) utilizes the strained alkyne derivatives as reactive partner to avoid the toxic catalyst.^[78]^ Nonetheless, the major drawbacks of SPAAC are the limited water solubility of strained alkyne and slow reaction kinetics.^[83]^ Strained cyclooctyne can also react with other 1,3‐dipoles, for example, nitrones, which is termed as “strain‐promoted alkyne‐nitrone cycloaddition (SPANC)”. SPANC reactions proceed rapidly with second order rate constants of up to 39 M^−1^ s^−1^, which is about 30 times faster than the SPAAC reaction.^[84]^ However, the fast reactivity is associated with the instability of the reactive nitrones, which are prone to hydrolysis in aqueous media.^[84]^ Photoclick reactions offer the advantages of operational simplicity as well as spatial and temporal control due to the usage of light.^[85]^ But recent evidences have shown that photoclick reactions could be limited by potential cross reactivity with, for example, amine residues, and its bioorthogonality still remains controversial.^[86]^ Among all the existing bioorthogonal reactions, the inverse electron demand Diels–Alder reaction (iEDDA) stands out because of the fast reaction kinetics (rate constant of up to 10^6^ M^−1^ s^−1^), high bioorthogonality, catalyst‐free conditions and good biocompatibility.^[87]^ Because of its very fast reaction kinetics, it revolutionized bioconjugation of various biomolecules and stimulated labeling in living systems with rate constants comparable to biological reactions.^[87]^ The currently available toolbox of diverse bioorthogonal reactions provides the basis for protein dual functionalization ensuring no cross‐reactivity and high yields of the bioconjugates. Figure 4 summarizes the different combinations of the bioorthogonal reactions that are commonly employed in the literature. Dual modification proceeds either in a sequential or simultaneous fashion. For example, if two identical CuAAC (or iEDDA) reactions are combined, protein dual modification has to be executed in a sequential manner to prevent cross‐reactions, which otherwise would result in a mixture of products. In contrast, the combination of CuAAC and iEDDA introduces two bioorthogonal tags on proteins simultaneously. Since both reactions allow quantitative conversions, dual‐modified bioconjugates can be obtained in a one‐pot reaction without the need for purification of the single modified product.^[82]^

## 3 Synthetic strategies for dual functionalization of proteins

Most synthetic strategies for protein dual modification target canonical AAs exposed at the protein surface. These residues are immediately accessible without the need for tedious genetic engineering of recombinant protein variants and also mitigate the risk of negative effects on proteins folding and function. In this section, dual functionalization at two different sites as well as at a single site is discussed.

### 3.1 Dual functionalization at two different sites

**Figure 5:**
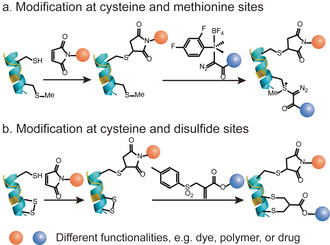
Site‐selective protein dual modification at different AA residues. (a) Cysteine and methionine sites.^[59]^ (b) Cysteine and disulfide sites.^[89]^

Direct modification of two rare AA residues on the protein surface is a straightforward approach to achieve dual modification of proteins. The combination of two different mono‐functionalization methods requires stringent selection to ensure orthogonality, compatibility and preferably high modification efficiency. For example, Gaunt and co‐workers developed a method based on chemoselective labeling of a single methionine residue with a hypervalent iodine reagent.^[59]^ This hypervalent iodine reagent selectively reacted with the moderate nucleophilic methionine residue in the presence of other competitive nucleophilic AA residues, which make it compatible and complementary to other bioconjugation strategies targeting other AA residues. This has been demonstrated by first modifying the unpaired cysteine residue of GTP‐binding protein fragment Gα with a maleimide derivative in a Michael reaction to form a thioether bond. Subsequent modification with hypervalent iodine reagent selectively addresses the thioether in a methionine residue yielding the dual modified protein bioconjugate (Figure 5 a). Notably, the methionine modification showed high site selectivity without any cross reactivity with the thiol–maleimide conjugation. Besides that, Paavola and co‐workers have also achieved dual functionalization by combining cysteine modification and an N‐terminal transamination reaction mediated by pyridoxal 5‐phosphate.^[88]^ The periplasmic glutamine binding protein was site‐selectively functionalized by a FRET pair, in which the ligand‐induced conformational movements were monitored via changes in FRET efficiency.

An alternative strategy for site‐selective protein dual modification is based on the differences in the reactivity of cysteines in their free (thiol) and oxidized (disulfide) forms.^[89]^ Disulfide bonds could be considered as protected thiols, which require activation to form the reduced free thiols for subsequent functionalization. Therefore, site‐selective dual modification of native proteins proceeds stepwise by modifying an unpaired cysteine with a maleimide reagent as the initial step, followed by the disulfide reduction to liberate two additional free thiols, which will react with a disulfide rebridging reagent, for example, an allyl sulfone (Figure 5 b). It is essential that the thiol–maleimide reaction should be applied first to functionalize the unpaired cysteine residue, followed by the disulfide functionalization using allyl sulfone reagents. Otherwise, the allyl sulfone reagents will also react with the unpaired cysteine, resulting in a heterogeneous product mixture.

### 3.2 Dual functionalization at a single site

Despite the simplicity of dual modification at two distinct sites, the availability of proteins with two different AA residues with orthogonal reactivities is rather limited. Therefore, alternative methods have been developed to target one specific AA residue with a multifunctional bioconjugation reagent.

**Figure 6:**
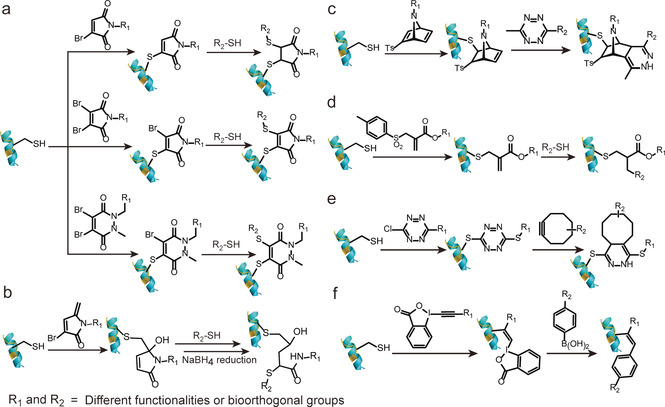
Site‐selective protein dual modification at a single cysteine site to introduce multifunctional bioconjugation reagents. (a) Mono and dibromomaleimide, dibromopyridazinediones (from top to down)[^47^, ^90^] (b) 3‐Bbromo‐5‐methylene pyrrolones (3Br‐5MPs)^[91]^ (c) Azabicyclic vinyl sulfone^[37]^ (d) Allyl sulfone^[38]^ (e) Dichloro‐1,2,4,5‐tetrazine^[44]^ (f) Ethynylbenziodoxolones (EBXs).^[46]^

Baker, Caddick, and co‐workers have demonstrated protein dual modification using mono‐ and dibromomaleimide reagents.^[47]^ The first functionality is introduced by reacting these reagents with an accessible thiol group through an addition‐elimination reaction. Subsequently, an additional thiol conjugation introduces the second functionality (Figure 6 a). A conceptually similar strategy utilizing dibromopyridazinediones is depicted in Figure 6 a.^[90]^ Interestingly, the native protein, for example, Grb2 adaptor protein, could be regenerated from the dual‐modified conjugate after addition of phosphine or a large excess of thiols, which opens access to the reversible modulation of proteins function or controlled release of the attached cargos, such as drug molecules.^[90]^ In a recent example, another maleimide analogue, 3‐bromo‐5‐methylene pyrrolones (3Br‐5MPs), was reported for cysteine‐specific dual modification of proteins, which has comparable modification efficiency but higher cysteine specificity than the traditional maleimide reagents (Figure 6 b).^[91]^ The dual modification was achieved by two sequential Michael reactions. First, a Michael reaction of cysteine and 3Br‐5MPs generated the bioconjugate that is amenable to a second Michael addition with another thiol, allowing protein dual functionalization at a cysteine site. Due to the slow release of the second functionality, a reducing reagent, for example NaBH_4_, was required to retard the elimination reaction to generate a stable and bioactive conjugate for subsequent applications.^[91]^

Alternatively, vinyl sulfones are also commonly explored Michael acceptors for protein modification due to its high electrophilic properties that enable their reaction with nucleophiles on the protein surface.^[92]^ Nevertheless, their application for dual functionalization was hampered by the cross‐reactivity with e.g., amino or imidazole groups generating heterogeneous products.^[93]^ Recently, Bernardes and co‐workers combined the strained [2.2.1]bicyclic systems with the vinyl sulfone systems and developed the azabicyclic vinyl sulfone reagents for dual functionalization (Figure 6 c).^[37]^ Such combination results in a fast chemoselective protein modification at the cysteine site, while the dienophile in the azabicyclic strained moiety concomitantly offers an opportunity for further bioorthogonal modification via iEDDA to liberate the energy stored in the strain systems. The second functionalization could even proceed inside living cells for selective apoptosis imaging. Besides vinyl sulfone, allyl sulfone reagents with enhanced water solubility and higher reactivity have also been proposed as a viable strategy for dual modification in a stepwise fashion.^[38]^ By simply adjusting the pH, allyl sulfone reagents first reacted in a Michael reaction at pH 6 to attach the first functionality, yielding a conjugated ester system that reacted with the second thiol‐containing moiety at pH 8 to achieve dual functionalization of proteins (Figure 6 d).^[38]^

In addition to maleimide analogues and sulfone derivatives, other strategies have also been developed for dual‐modification. For example, Goncalves and co‐workers reported that dichloro‐1,2,4,5‐tetrazine can undergo two successive nucleophilic aromatic substitutions to introduce the thiol‐containing payload at a cysteine residue with excellent selectivity (Figure 6 e).^[44]^ The tetrazine linkage could serve as the second handle for subsequent bioorthgonal iEDDA reaction, allowing the preparation of site‐selective dual‐modified protein conjugates. The feasibility of this strategy has been shown by the dual labeling of the human serum albumin with a macrocyclic chelator for nuclear imaging and a fluorescent probe for fluorescence imaging.^[44]^ Despite the simplicity of this method, it could suffer from lower yield if bulky and hydrophobic functionalities needs to be incorporated. Furthermore, a different strategy utilizing the inherent reactivity of the hypervalent bond was also reported. Waser and co‐workers showed the dual modification of proteins with ethynylbenziodoxolones (EBXs) in high efficiency and chemoselectivity by introducing two reactive groups, i.e., an azide and a hypervalent iodine (Figure 6 f).^[46]^ Dual modification was achieved via a strain‐release‐driven cycloaddition and Suzuki–Miyaura cross‐coupling of the vinyl hypervalent iodine bond with using palladium diacetate complex as catalyst.

**Figure 7:**
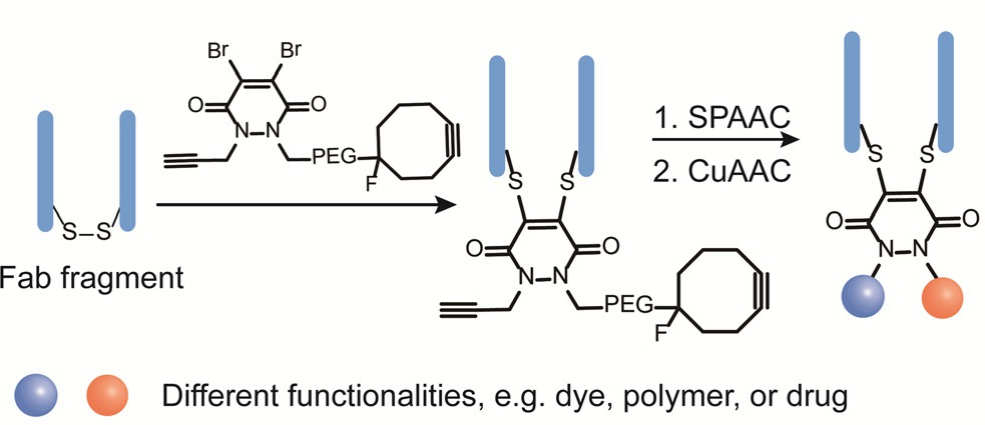
Site‐selective dual modification of proteins at the disulfide site of antibody Fab fragment.^[50]^

Due to the emergence of ADCs for targeted cancer therapy, dual functionalization at disulfide site has also gained growing interest because of the presence of accessible disulfide bonds in antibodies and the antigen‐binding fragment (Fab).[^11^, ^12^] Previously, dibromopyridazinediones have been extensively employed as versatile reagents for dual modification at the single cysteine site.^[90]^ Further reports revealed that it can also serve as a disulfide rebridging reagent to introduce two bioorthogonal tags into disulfide‐containing proteins, for example, antibodies or antibody fragments. Chudasama et al. exploited the insertion of dibromopyridazinediones bearing two bioorthogonal tags into the disulfide bonds in full antibody and antibody Fab fragments (the antigen‐binding fragment) without perturbing the internal disulfide bonds that are vital for activity (Figure 7).^[50]^ Such a plug‐and‐play platform allowed the introduction of two functionalities via two sequential bioorthogonal reactions in a modular and efficient way, paving the way for the next‐generation ADCs.

Besides addressing cysteines and disulfide bonds, other modification strategies at less‐explored AA residues have also been reported to expand the existing protein functionalization toolkit. For example, the hypervalent iodine reagents developed by Gaunt and co‐workers can be combined with maleimide reagents to achieve dual functionalization at cysteine and methionine sites, which is described in Figure 5 a. In addition, the hypervalent iodine reagents have also been demonstrated to show multifaceted reactivity.^[59]^ The electrophilicity of the diazo sulfonium conjugate enables a photoredox radical cross‐coupling reaction with C‐4 benzylated Hantzsch ester derivatives to attach the second functionality yielding dual functionalized conjugates with high conversion.^[59]^

Achieving dual modification at one single AA site has less restriction in terms of the choice of reagents compared to the functionalization at two AA sites. One possible limitation of the single site strategy is that the two orthogonal groups can be sterically hindered due to close proximity on a relatively small multifunctional linker. This could prevent bulkier groups such as polymers to be attached onto the protein. In addition, modification besides cysteine residues is relatively unexplored, thus this field would greatly benefit from further investigations of modification strategies at other low abundant AA residues such as tyrosine, serine or the N‐termininus.

## 4 Genetic engineering for dual functionalization of proteins

To expand the reactivity beyond what is offered in native proteins, reactive groups for functionalization through genetic incorporation of new AAs (canonical or noncanonical ones) has emerged as an indispensable tool for site‐selective protein dual functionalization. In this section, genetic methods to integrate new reactive canonical AAs, ncAAs or peptides tags for protein dual functionalization are summarized.

### 4.1 Incorporation of canonical AAs

**Figure 8:**
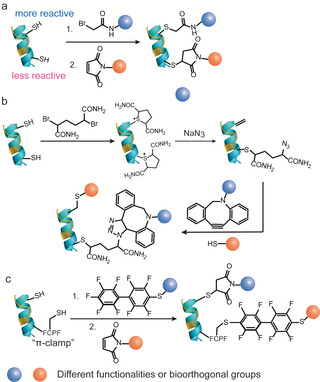
(a) Genetic encoding of two cysteine mutations with different nucleophilicity.^[94]^ (b) Genetic encoding of two cysteine mutations possessing different protein local microenvironment.^[95]^ (c) Genetic encoding of a cysteine mutation and a “π‐clamp” FCPF peptide sequence creates a new microenvironment for the cysteine residue.^[96]^

The expression of recombinant proteins containing one or more point mutations is a straightforward and well‐established technology.^[84]^ Early attempts to achieve dual functionalization of proteins via genetic methods focused on the preparation of dual cysteine mutants of the target protein. Two cysteine mutations were incorporated at two distinct locations, and both thiols exhibited different reactivity toward different thiol‐reactive reagents.^[94]^ For instance, Caddick et al. reported the two‐cysteine insertions to genetically engineered antibody mimetic proteins, so‐called designed ankyrin repeat proteins (DARPins).^[94]^ Both thiols are carefully selected and revealed different nucleophilicity, which may origin from their different solvent accessibility. This allowed for the dual functionalization to be executed in a stepwise fashion with high overall labeling efficiency (Figure 8 a).^[94]^ The authors proposed that the less reactive reagent (bromoacetamide) was reacted first with the more nucleophilic cysteine and subsequently, the more reactive reagent (maleimide) was applied to the second, less nucleophilic cysteine. They successfully demonstrated that the differences in thiol nucleophilicity afforded a homogeneous product with quantitative conversion at each reaction step, and no purification was needed. Although this concept for dual modification of proteins is very elegant, the delicate balance of thiol reactivity and the selection of the most appropriate cysteine mutation site to prevent heterogeneous product formation (single and dual modified products) could be very challenging. Caddick and co‐workers also succeeded in protein dual modification by reacting two cysteines with the same thiol‐reactive reagent generating two identical sulfoniums.^[95]^ Due to the different accessibilities of the α‐protons at the two cysteine mutations, one of the sulfonium groups, which had a good solvent accessible α‐proton on adjacent position, underwent a β‐elimination reaction affording dehydroalanine (Figure 8 b). In contrast, the other sulfonium group, which has a shielded α‐proton next to it, remained intact because of the different protein local microenvironment. The functionalities were incorporated via two different chemoselective reactions, offering the site‐selective dual‐modified protein conjugate in high yield.

The examples mentioned above took advantage of the different local microenvironment offered by the native protein. Inspired by Nature's elegance, Pentelute and co‐workers have developed a strategy to create a specific local chemical environment for the cysteine residue by a newly developed, fine‐tuned four‐amino‐acid peptide sequence (FCPF), which is termed as “π‐clamp” (Figure 8 c).^[96]^ This π‐clamp enables the conjugation exclusively at this cysteine site with perfluoroaromatic reagents with almost quantitative conversion. The reaction proceeds even in the presence of other competing thiols, thus rendering this approach compatible and complementary to other thiol‐conjugation strategies.^[96]^ Dual functionalization was demonstrated on a model protein substrate bearing a cysteine and a π‐clamp mutation, which was functionalized with a perfluoroaryl probe first based on the π‐clamp‐mediated conjugation and followed by the thiol–maleimide conjugation reaction.^[96]^

### 4.2 Incorporation of ncAAs

**Figure 9:**
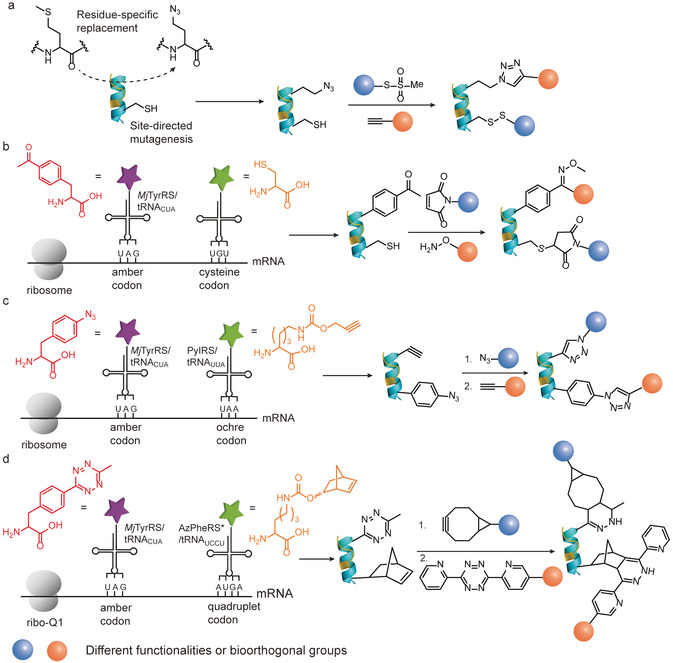
(a) Site‐selective incorporation of one cysteine mutation via site‐directed mutagenesis and one methionine analogue via residue‐specific replacement experiment.^[98]^ (b) Genetic encoding of one cysteine mutation via site‐directed mutagenesis and one ncAA via genetic code expansion in response to UAG codon.^[60]^ (c) Genetic encoding of two noncanonical AAs via genetic code expansion in response to UAG and UAA codon.^[100]^ (d) Genetic encoding of two noncanonical AAs via genetic code expansion in response to UAG and AUGA codon.^[25]^

The introduction of point mutations has certain limitations as the available functionalities and their respective reactivities could only be selected from the pool of the 20 canonical AAs. However, the cellular biosynthetic machinery can be manipulated to incorporate ncAAs that often represent structurally similar derivatives of the canonical AAs. These ncAAs allow protein labeling with unprecedented molecular precision.^[97]^ To date, a diverse set of ncAAs with various functionalities or bioorthogonal groups has been reported for genetic incorporation into proteins.[^3^, ^84^] In the auxotrophic strain, organism such as *E. coli* are applied that are not able to synthesize a certain amino acid required for its growth. The ncAAs need to structurally resemble the natural AA to allow binding to the respective endogenous aminoacyl‐tRNA synthetase and to replace the natural AA in the polypeptide sequence.^[84]^ This strategy has been mainly employed to introduce azide‐ or alkyne‐containing methionine analogues in a methionine‐auxotrophic *E. coli* strain.^[84]^ For example, the Davis group incorporated the methionine analogue, azidohomoalanine (AHA), and a cysteine mutation to the target protein based on the combination of site‐directed gene mutagenesis and the residue‐specific replacement of methionine by its analogues (Figure 9 a).^[98]^ Dual modification was accomplished through the initial cysteine conjugation with methanethiosulfonates derivatives, followed by the CuAAC reaction of the azido group in AHA and ethynyl functionalities. This approach offers the benefit of the established AHA incorporation and its efficient modification by cycloaddition reactions. However, the set of available ncAA is limited as they have to bind to their respective tRNA synthetase efficiently, using auxotrophic strains and all the AA residues within a sequence will be replaced by the respective ncAA analogue.

The genetic code expansion technique has been developed as another technique that allows the insertion of a broad variety of ncAAs with spatial precision at virtually any desired site.^[97]^ It is accomplished by using an orthogonal aminoacyl‐tRNA synthetase (aaRS)/tRNA pair, which is capable of charging a designed ncAA in response to a nonsense codon, such as the amber stop codon (UAG), due to their minimal occurrence in most organisms.[^97^, ^99^] This strategy allows the genetic encoding of more than 150 ncAAs containing various reactive handles and functionalities.^[84]^ Therefore, the incorporation of ncAA via genetic code expansion in combination with the site‐directed canonical AA mutation provides a versatile strategy for protein dual functionalization. Representative work was reported by Deniz and co‐workers, in which they utilized an engineered tyrosyl‐tRNA synthetase (MjTyrRS)/tRNA_CUA_ pair (*Mj*TyrRS/tRNA_CUA_ pair) derived from *Methanococcus jannaschi* in response to the amber (UAG) stop codon to insert *p*‐acetylphenylalanine, a ketone bearing ncAA, into T4 lysozyme.^[101]^ In combination with a single cysteine mutation, dual labeling of the T4 lysozyme mutant was demonstrated by modification with a FRET dye pair through the thiol–maleimide reaction and oxime ligation (Figure 9 b).^[101]^

Despite the simplicity of introducing a cysteine mutation as one of the target sites for protein dual modification, this approach could become problematic if the protein consists of native cysteines that play important structural or functional roles. Furthermore, cysteine residues could form disulfide bonds, which could limit expression yields and the reaction reversibility of the thiol–maleimide conjugation could also cause stability problems of the resulting protein bioconjugates.^[36]^ Hence, there have been much efforts to introduce two different ncAAs with bioothogonal tags that could be functionalized independently. Liu and co‐workers applied two mutually orthogonal aaRS/tRNA pairs in response to two blank codons.^[100]^ They mutated the pyrrolysyl‐tRNA synthetase (PylRS)/tRNA_CUA_ pair (PylRS/tRNA_CUA_ pair) to produce a new variant, PyIRS/tRNA_UUA_, which can suppress the ochre (UAA) stop codon. In combination with the evolved *Mj*TyrRS/tRNA_CUA_ pair, these two orthogonal aaRS/tRNA pairs were capable of recognizing and inserting two ncAAs, *p*‐azido‐l‐phenylalanine and N^*ϵ*^‐propargyloxycarbonyl‐l‐lysine, into a glutamine‐binding protein in response to the amber UAG codon and the ochre UAA codon (Figure 9 c).^[100]^ Two sequential CuAAC reactions were employed to install two chromophores for the protein dual modification. In a subsequent report, the authors incorporated the azide‐ and ketone‐bearing ncAAs into GFP with the use of an evolved *Mj*TyrRS (AzFRS)/tRNA_CUA_ pair and PyIRS (AcKRS)/tRNA_UUA_ pair, to eliminate protein aggregation and oxidation induced by the copper catalyst.^[81]^ Notably, dual functionalization was accomplished by SPAAC and an oxime ligation in one‐pot and catalyst‐free fashion. In addition, besides in *E. coli*, Schultz and co‐workers have also successfully incorporated two different ncAAs, which contained azido and ketone groups, in mammalian cells by utilizing the two orthogonal *Methanosarcina barkeri* pyrrolysyl‐tRNA synthetase (MbPylRS)/*Methanosarcina mazei* pyrrolysyl tRNA (*Mb*PylRS/*Mm*tRNA_UUA_ pair) and tyrosyl‐tRNA synthetase (EcTyrRS)/tRNA_CUA_ pair (*Ec*TyrRS/tRNA_CUA_ pair).^[102]^ The dual‐tagged antibody was subsequently functionalized with a toxic drug payload and a fluorophore with high conversion.

Alternatively, instead of reassignment of the triplet stop codons, Chin and co‐workers have exploited the two orthogonal aaRS/tRNA pairs in response to a quadruplet blank codon (four‐base codon) and a stop codon for the incorporation of two ncAAs.^[103]^ However, natural ribosomes suffer from very low efficiency in decoding the quadruplet codon. In this context, Chin et al. have synthetically evolved an orthogonal lysozyme (ribo‐Q1), which was not responsible for synthesizing the proteome as natural lysozyme, for selectively decoding the quadruplet codon.^[79]^ By the combination of ribo‐Q1 with two orthogonal aaRS/tRNA pairs, AzPheRS*/tRNA_UCCU_ (a derivative of *Mj*TyrRS/tRNA) and PylRS/tRNA_CUA_, two ncAAs were site‐selectively introduced into Calmodulin, forming a triazole intramolecular crosslink through the subsequent CuAAC reaction.^[79]^ Nonetheless, the major drawback of this system lies in the low efficiency and specificity of the AzPheRS*/tRNA_UCCU_ pair in directing the corresponding ncAA incorporation. The efficiency of the original system was substantially improved based on the evolution of the PylRS/tRNA_CUA_ to obtain an optimized quadruplet decoding variant, PylRS/tRNA_UACU_ pair.^[25]^ As a proof‐of‐concept, the evolved PylRS/tRNA_UACU_ pair was combined with the AzPheRS*/tRNA_CUA_ pair to site‐selectively introduce two bioorthogonal tags, norbornene and tetrazine, into Calmodulin (Figure 9 d).^[25]^ Dual modification was successfully accomplished via two sequential iEDDA reactions.^[25]^ With the established technologies, in the latter example, the authors incorporated the alkyne and cyclopropane handles into Calmodulin by using the orthogonal aaRS‐tRNA pair described above, permitting the simultaneous dual functionalization in one pot.^[82]^

Genetic code expansion has witnessed incredible achievements in recent years, which greatly promotes and revolutionizes the field of site‐selective protein dual functionalization. The advantages of this strategy lie in the small size of the ncAAs, flexible incorporation sites but high site‐selectivity, and the diverse reactive tags available for incorporation. However, some important challenges still remain in this rapidly evolving field, for example, low catalytic efficiency of engineered aaRS which require tedious evaluation and optimization to improve their performance, relative low expression yields, repetitive optimization of the protocols for effective protein engineering, the scope and compatibilities of the functional groups capable to be inserted.^[84]^ Besides developing the recombinant engineering techniques, the bioorthogonal chemistry toolbox can also be expanded so that there are more choices for compatible bioorthogonal reaction pairs in terms of reaction rate, catalyst type, and substrate solubility for their intended applications.

### 4.3 Incorporation of peptide tags

**Figure 10:**
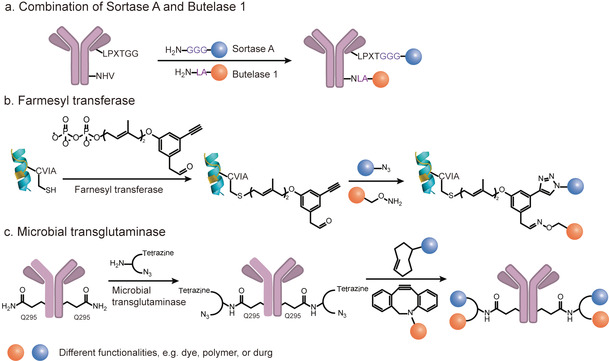
Dual modification of proteins by using (a) the combination of Sortase A and Butelase A.^[118]^ (b) Farmesyl transferase through the incorporation of a dual‐functional scaffold bearing an alkyne and an aldehyde tag.^[107]^ (c) Microbial transglutaminase through the incorporation of a dual‐functional scaffold bearing a tetrazine and an azido tag.^[80]^

Besides the incorporation of ncAAs bearing bioorthogonal tags for dual modification of proteins, the insertion of two artificial peptide sequences that can be recognized by two distinct enzymes for the subsequent covalent labeling with user‐defined probes also serves as a straightforward approach for dual modification of proteins. This enzyme‐mediated peptide labeling combines the advantage of high specificity of the enzyme towards the peptide tags and excellent labeling efficiency with minimal perturbation to proteins structure and function.^[104]^ A brief summary of the commonly used peptide tags and their respective enzymes is given in Table 2 and relevant examples for dual modification are further discussed in this section. In a recent example, two distinct enzymes, Sortase A and Butelase 1, which demonstrate orthogonal specificity to LPXTGG and HNV motif, respectively, have been combined for dual modification of IgG antibody (Figure 10 a).^[118]^ Notably, a simple centrifugation process is sufficient to obtain the pure dual‐modified protein conjugate, demonstrating the high selectivity and excellent conversion of the enzyme‐mediated labeling approach. In addition, peptide tags have also been utilized in combination with ncAAc^[105]^ or “π‐clamp” sequence^[106]^ for site‐selective dual‐modification of proteins. For example, Chen and co‐workers have demonstrated the incorporation of a LAP peptide (LplA acceptor peptide), which can be recognized by a lipoic acid ligase (LpIA) to ligate a lipoic acid derivative, and an pyrrolysine analogues which bear an azido group for the site‐selective dual labeling of epidermal growth factor receptor (EGFR) on living cells.^[105]^

**Table 2:** Commonly used peptide tags and their respective enzymes.

Tag sequence^[a]^	Enzyme
GLNDIFEAQKIEWHE^[108]^	Biotin ligase^[108]^
LPXTGG^[109]^	Sortase A^[109]^
NHV^[110]^	Butelase A^[110]^
CaaX^[b][111]^	Farmesyl transferase^[111]^
LLQG^[112]^	Microbial transglutaminase^[112]^
GFEIDKVWYDLDA^[113]^	Lipoate acid ligase A^[113]^
CXPXR^[114]^	Formylglycine Generating Enzyme^[114]^
GDSLSWLLRLLN^[115]^	Phosphopantetheinyl transferase^[115]^
VDSVEGEGEEEGEE^[116]^	Tubulin tyrosine ligase^[116]^
AHIVMVDAYKPTK(Spytag)^[117]^	SpyCatcher^[117]^

[a] The capital letters are the abbreviation of different amino acids. “X” represents any amino acids. [b] “a” represents small aliphatic amino acids and “X” denotes one of Ala, Ser, Met or Glu residues amino acids in CaaX motif.

Despite the simplicity of a one‐step introduction of the desired functionalities by two independent reactions, the labeling efficiency may be greatly influenced by the steric effects and hydrophobicity of the introduced probes. This limitation can be overcome by the incorporation of a multifunctional scaffold possessing the bioorthogonal tags. Distefano et al. used farnesyl transferase, which can catalyze the transfer of an isoprenoid group from farnesyl diphosphate to the cysteine site of a tetrapeptide (CVIA, the letter code is the abbreviation of a specific AA), to introduce two reactive tags to the model protein, GFP (Figure 10 b).^[107]^ The bifunctional alkyne‐aldehyde modified protein can undergo two independent bioorthogonal reactions simultaneously, offering the dual‐modified conjugate in good conversion.

In some instances, the enzyme‐mediated dual labeling of proteins can also be achieved on the native protein surface in a site‐selective manner without the need to insert artificial peptide tags. For example, in Alabi and cowokers’ work, microbial transglutaminase, which catalyzes the formation of interprotein isopeptide bonds between glutamine and lysine residues, can recognize the glutamine 295 on the aglycosylated human IgGs and thus allowed the incorporation of two bioorthogonal tags, azido group and methyltetrazine group (Figure 10 c).^[80]^ The combination of SPAAC and iEDDA enables the generation of a dual‐modified antibody therapeutic containing a cytotoxic payload along with a hydrophobicity‐masking PEG side chain in a mix‐and‐match manner. Nevertheless, this strategy is greatly limited by the protein substrate, which requires the recognition motif to be present on the native protein surface at a specific site.

## 5 Higher‐level functionalization of proteins

Driven by exciting developments in the discovery of new synthetic procedures as well as scientific curiosity, achieving higher level of protein modification in a site‐selective manner is considered crucial for advancing different fields in chemistry, biology and material science. Compared to mono‐ and dual functionalization of proteins, triple functionalization strategies allow the incorporation of three different functionalities into proteins, thus offering well‐defined protein bioconjugates with further expanded structural and functional diversities that is beyond what current synthetic strategies can accomplish. These endeavors will greatly boost our capability to investigate, modulate and re‐design the chemical and physical properties of proteins.

However, triple functionalization of proteins is even more demanding than mono‐ and dual functionalization as the combination of three orthogonal chemistries have to be applied in aqueous solution and in the presence of many reactive proteinogenic groups. Furthermore, by increasing the number of functionalities attached to the protein surface, it may lead to potential detrimental influence to their functional and structural integrity. In addition, the selection of the reactive groups requires more deliberate chemical design as the increase in the hydrophobicity of the bioconjugation reagents may cause protein aggregation. The labeling efficiency may also be compromised due to the steric hindrance from the insertion of three different functionalities.

**Figure 11:**
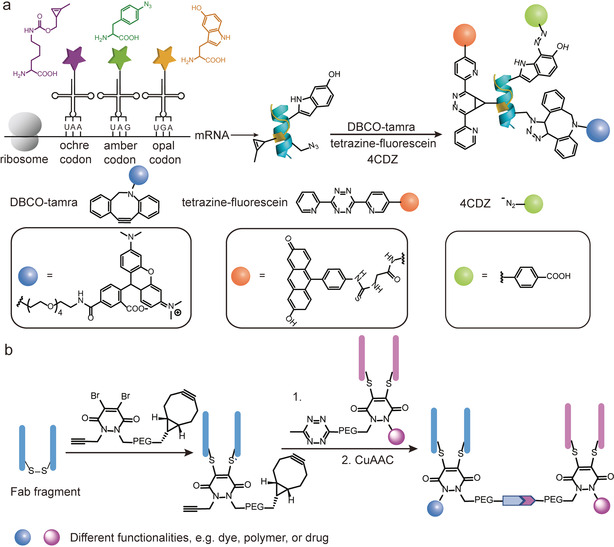
(a) Genetic incorporation of three ncAAc into protein via genetic code expansion in response to the UAA, UAG and UGA codons for tri‐functionalization of proteins.^[119]^ (b) Preparation of antibody bispecifics by the conjugation of two dual‐modified antibody Fab fragments.^[122]^

Recent examples have demonstrated the successful triple functionalization of proteins. Chatterjee and co‐workers have reported the introduction of three ncAAs into proteins via genetic code expansion. In this work, they assigned the EcTrp, *Mj*Tyr, and Pyl pairs to suppress UGA, UAG and UAA codons for the incorporation of three ncAAs, 5‐hydroxytryptophan, *p*‐azidophenylalanine, and cyclopropene‐lysine, in the engineered *E. coli* strain ATMW1 (Figure 11 a).^[119]^ The triple functionalization of the protein was successfully achieved by three mutually compatible reactions, SPAAC, iEDDA, and chemoselective rapid azo‐coupling reaction (CRACR), in which the electron‐rich 5‐hydroxyindole ring in 5‐hydroxytryptopan reacts with electron‐deficient aromatic diazonium ions with fast reaction kinetics and high conversion.^[120]^ This is the first example of triple modification of proteins where three ncAAs were incorporated into the target protein in living cells, which further expands the simultaneous ncAAs coding capacity via genetic code expansion. However, this strategy utilized three different aaRS/tRNA pairs and three stop codons, which leaves no codon for termination of endogenous genes and therefore greatly interferes with the translation termination inside cells. Shortly after, Chin and co‐workers have demonstrated the genetic encoding of three distinct ncAAs into proteins with three aaRS/tRNA pairs in a different strategy.^[121]^ In their work, they identified new ΔNPyIRS/^ΔNPyI^tRNA pairs, which lack the N‐terminal domains, and revealed that they can be assigned into two classes, class A and B, based on their sequences. Specifically, class A ΔNPyIRS preferentially function with class A ^ΔNPyI^tRNA and vice versa. Next, they discovered a *Mm*PyIRS/*Spe* ^PyI^tRNA pair, in which *Spe* ^PyI^tRNA is orthogonal to both class A and B ΔNPyIRS. In this context, the triply orthogonal aaRS/tRNA pairs were identified, which contained the *Mm*PyIRS/*Spe* ^PyI^tRNA pair, an evolved class A ΔNPyIRS/^ΔNPyI^tRNA pair and an evolved class B ΔNPyIRS/^ΔNPyI^tRNA pair. Three ncAAs, N*ϵ*‐((tert‐butoxy)carbonyl)‐l‐lysine, 3‐methyl‐l‐histidine and N*ϵ*‐(carbobenzyloxy)‐l‐lysine, were genetically encoded into proteins in response to the UAG, AGGA and AGUA codons. However, subsequent functionalizations have not demonstrated yet. Compared to the reassignment of three stop codons, this work utilizes the combination of a stop codon and quadruplet codons, which do not interfere with the translation termination and serve as a more general and applicable strategy to achieve higher‐level protein functionalization. Even though there is no application shown and there are still obvious limitations, for example, low encoding efficiency, these examples represent the first proof‐of‐concept for protein triple modifications. They still represent significant technological advancements for the development of multifunctional protein conjugates, which holds immense potential for drug delivery, bioimaging, and material science.

Besides increasing the number of functionalities that can be incorporated into proteins, more complex protein bioconjugates have also been reported. For example, Chudasama et al. described an efficient and modular strategy for the generation of a bispecific antibodies as well as the dual functionalization of the resulting bioconjugate (Figure 11 b).^[122]^ By fine‐tuning the bioorthogonal chemistry employed, two dual‐modified antibodies were first conjugated together via the iEDDA reaction of the two bioorthogonal handles from each antibody (Figure 11 b). Subsequently, two other bioorthogonal handles from each antibody allow for the dual functionalization of the chemically constructed bispecific antibodies. Despite these seminal studies, the preparation of protein conjugates with higher level of modification or structural complexity remains relatively unexplored. We envision that with technical breakthrough in methodology, exciting yet unexplored applications will be discovered, which will undoubtedly revolutionize fundamental studies and applications of proteins.

## 6 Applications

With the emergence of protein dual functionalization techniques, homogeneous protein bioconjugates with remarkable functional complexity have been achieved and studied for diverse applications. In the following, three major fields of applications are highlighted that have greatly benefited from these novel synthesis opportunities.

### 6.1 Probing protein dynamics through Förster resonance energy transfer

The excitation energy transfer from a donor to an acceptor chromophore, also termed Förster resonance energy transfer (FRET), allows assessing important parameters such as chromophore distances and their orientations in real time and in complex biological environments. Although cryo‐electron microscopy is becoming a popular technique to determine structural snapshots of biomacromolecules at atomic resolution, samples need to be frozen and studies cannot be carried out in native environment. In contrast, FRET bears the advantage of easy accessibility and enables the real‐time monitoring of the dynamics and conformational changes of proteins in their native environment, thus serving as an important complementary methodology for elucidating proteins structural dynamics.^[123]^ A critical requirement for FRET studies is the site‐selective incorporation of the respective donor and acceptor chromophores at pre‐defined locations on the protein surface without interfering with the proteins’ structure and function.^[123]^

**Figure 12:**
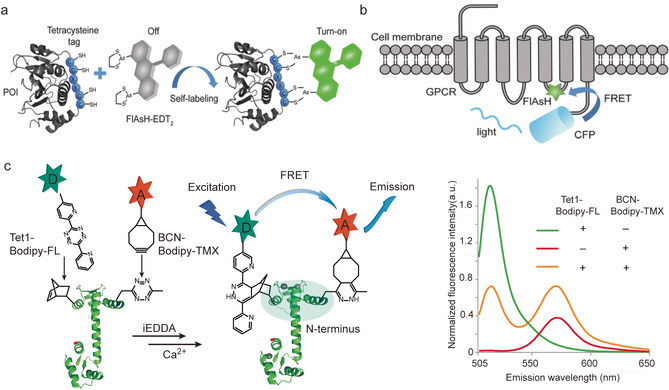
(a) The self‐labeling peptide of tetracysteine reacting with FlAsH forming fluorescent conjugate. Adapted with permission from ref. [15]. Copyright (2015) Royal Society of Chemistry (b) Incorporating the chromophore and CFP as FRET pair to GPCR to probe protein dynamics. Adapted with permission from ref. [15]. Copyright (2015) Royal Society of Chemistry (c) Incorporation of FRET pair into Calmodulin (CaM) via genetic code expansion technique and the corresponding fluorescence spectra after addition of Ca^2+^. Adapted with permission from ref. [25]. Copyright (2014) Springer Nature.

Fluorescent proteins (FPs), for example, cyan fluorescent protein (CFP) and yellow fluorescent protein (YFP), can be genetically encoded into the target proteins and are widely utilized for the investigation of proteins inside living systems.^[15]^ For example, agonist‐induced activation of G‐protein coupled receptor (GPCRs) is thought to cause a conformational rearrangement of their seven transmembrane α‐helices.^[124]^ The insertion of CFP and YFP to the G‐protein coupled receptors (GPCR) offered the dual tagged protein bioconjugate, which allows real‐time monitoring of the activation switch of GPCRs in living cells.^[125]^ However, due to the large size of FPs (molecular weight close to 30 kDa), the CFP‐YFP FRET pair was found to perturb the proteins structure and function and impair the downstream signal transduction.^[125]^ In order to overcome these limitations, the self‐labeling peptide tags have been developed, which can bind or react with small‐molecule reagents to attach the fluorophore to target proteins. For example, tetracysteine‐containing motif CCXXCC (C is the abbreviation of cysteine and XX can be virtually any AA sequence), can selectively react with fluorescein arsenical hairpin binder (FlAsH) to form fluorescent product (Figure 12 a).^[126]^ Lohse and co‐workers used the FlAsH‐tetracysteine system to label the human adenosine A_2A_ receptor in a site‐specific fashion, which, in conjunction with a CFP, generated a FRET construct (A_2A_‐FlAsH‐CFP) that exhibited a 5‐fold enhanced agonist‐triggered FRET signal compared to the dual‐tagged CFP and YFP conjugate (Figure 12 b).^[127]^

In comparison to FPs, small molecule chromophores bear the advantage of small size, excellent photostability and high quantum yield.^[128]^ So the incorporation of two chromophores into target proteins as FRET pair have represented as a more advantageous strategy to probe protein dynamics without interfering their structure and functions. One representative example is the introduction of two fluorophore into a calcium‐binding protein, Calmodulin (CaM), via genetic code expansion to study its conformation changes as a function of the calcium ion (Ca^2+^) concentration. CaM consists of two domains, the C‐terminal and N‐terminal domain that contain two EF‐hand motifs to bind Ca^2+^.^[129]^ Upon Ca^2+^binding, the conformation of CaM changes, which affects its binding to different target enzymes.^[129]^

The structures of CaM with four Ca^2+^ and without Ca^2+^ have been resolved in detail via NMR or X‐ray crystallography. However, the transition states that dynamically occur during the Ca^2+^ binding processes and that play an important role in modulating the interaction of CaM with its binding partners could not be resolved with conventional structure analysis by NMR and X‐Ray.^[130]^ Therefore, dual‐tagged CaM was obtained by genetic code expansion and conjugated with the FRET pairs, BODIPY‐TMR‐X (Bodipy‐tetramethylrhodamine, acceptor) and BODIPY‐FL (4,4‐difluoro‐5,7‐dimethyl‐4‐bora‐3a,4a‐diaza‐s‐indacene‐3‐propionic acid, donor), respectively, to investigate the local conformation changes occurring in the N‐terminal domain of CaM in response to the Ca^2+^ concentration (Figure 12 c).^[25]^ After excitation at 485 nm, the dual chromophore tagged CaM reveals two distinct signal corresponding to the emission of the BODIPY‐FL donor at 515 nm and the BODIPY‐TMR‐X acceptor at 570 nm. With increasing Ca^2+^ concentrations, the emission intensity of the acceptor dye decreased while the emission of donor dye increased indicating that the FRET pair at the N‐terminal domain moved apart as a response of Ca^2+^ binding. The current applications utilizing FRET to follow protein dynamics are mainly applying a certain stimulus in test tubes rather than measurements inside cells. In the future, continuous advancements in dual modification of proteins could provide valuable tools to shed light on the dynamics of proteins in living cells or even in organisms. There is much interest and progress in probing protein labeling reactions proceeding in living environments. However, no dual labeling reactions have been achieved inside cells that would allow FRET studies until now.

### 6.2 Combination therapy

ADCs have emerged as one of the most powerful and promising strategies for targeted cancer therapy.^[12]^ This new class of biopharmaceuticals combines the exquisite target‐specificity of antibodies with a highly potent cytotoxic payload via various conjugation technologies and enables the selective delivery of the payload to cancer cells with minimal off‐target effects.[^11^, ^12^] Owing to the elegant concept, there are already four ADCs approved by the US Food and Drug Administration on the market and over 60 ADCs are in clinical trials, demonstrating its great prospect for targeted therapeutic applications.^[12]^

**Figure 13:**
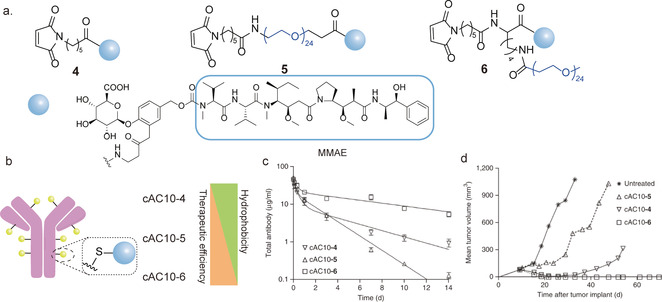
(a) Chemical structure of the three different drug linkers (**4**, **5** and **6**). (b) Preparation of the corresponding ADCs (cAC10‐4, cAC10‐5 and cAC10‐6). (c) Plasma pharmacokinetics in rats of cAC10 ADCs (cAC10‐4, cAC10‐5 and cAC10‐6), (data are given as means ± standard deviations.; *n*=3 animals per group). (d) Antitumor activity of cAC10 ADCs in the Hodgkin's lymphoma model L540cy (data are given as means; *n*=6 animals per group) at a single dose of 1 mg kg^−1^ (cAC10‐4 and −6) or 2 mg kg^−1^ (cAC10‐5). (c, d) Adapted with permission from ref. [132]. Copyright (2015) Springer Nature.

Despite the clinical success, most ADCs are still restricted to the conjugation with a single type of payload, for example a certain anticancer drug, to target a specific type of cancer cells. In fact, this kind of single‐functionalized ADCs often suffer from the unfavorable pharmacokinetics and the inherent hydrophobicity of the chemotherapeutic drugs, which could significantly limit the in vivo therapeutic efficiency.^[11]^ Dual modification of antibodies can serve as an elegant strategy to alleviate these drawbacks, allowing for the incorporation of two complementary modalities to achieve synergistic effects. This can be exemplified by the studies on extending the plasma circulation half‐life and attenuating immunogenicity of proteins through the attachment of hydrophilic polyethylene glycol (PEG) chain to proteins. The hydrodynamic radius of the biomolecule also increases after attaching a long PEG chain, thereby mitigating the renal filtration and circumventing its degradation by proteases and recognition by the immune system, which would otherwise lead to faster plasma clearance.^[131]^ In this context, dual modification of antibodies allows the simultaneous introduction of a cytotoxic drug molecule and a PEG chain in a site‐selective manner to afford well‐defined dual‐functional ADCs with improved pharmacokinetics. Senter and co‐workers have reported that the accelerated plasma clearance of ADCs originated from their hydrophobicity, which could be reduced by introducing a PEG chain with different configuration.^[132]^ As such, three different drug linkers were designed, in which the first drug linker **4** had no PEG chain while both the drug linker **5** and **6** consisted of a unfunctionalized PEG chain (PEG_24_,containing 24 repeating units) but with different configuration (Figure 13 a).^[132]^ Specially, drug linker **6** incorporated a branched scaffold. The cAC10 antibody (CD30‐directed antibody) was conjugated with the three different drug linkers via thiol–maleimide chemistry offering three different ADCs, cAC10‐4 (without PEG24), cAC10‐5 (with PEG24) and cAC10‐6 (with branched PEG24) as depicted in Figure 13 b.^[132]^ Both the in vitro and in vivo experiments revealed an inverse correlation between hydrophobicity and tumor volume (Hodgkin's lymphoma), namely that cAC10‐6 exhibited slower plasma clearance and much slower tumor growth compared to cAC10‐4 and cAC10‐5 (Figure 13 c,d).^[132]^ In particular, the improved pharmacokinetics and therapeutic efficiency of the branched conjugate cAC10‐6 compared to the linear conjugate cAC10‐5 highlights the importance of the hydrophilic linkers for optimizing the pharmacokinetic parameters.

**Figure 14:**
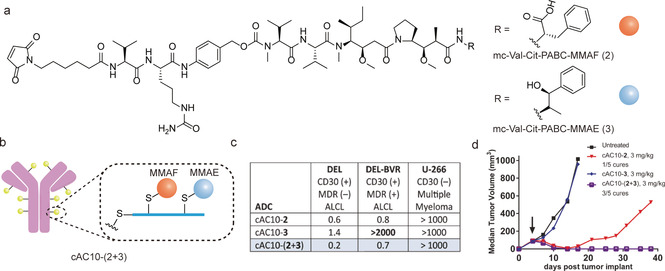
(a) The chemical structure of two different drug linker (mc‐Val‐Cit‐PABC‐MMAF and mc‐Val‐Cit‐PABC‐MMAE). (b) ADCs with the incorporation of two different drug molecules: MMAE and MMAF. (c) *In vitro* data of antitumor activity of cAC10‐(2+3) on MDR(+)DEL‐BVR cell lines. The values are reported as IC_50 ng_/mLof ADC. ALCL=anaplasticlarge cell lymphoma. MDR=multidrug resistance. DEL‐BVR is a cAC10‐vc‐MMAE resistant cell line, which is generated after prolonged exposure of the DEL anaplastic large cell lymphoma (ALCL) cell line to cAC10‐3 (d) In vivo data of antitumor activity of cAC10‐(2+3) on MDR(+)DEL‐BVR cells lines. (c, d) Adapted with permission from ref. [27] under the Creative Commons CC BY‐NC license. Copyright (2017) Wiley‐VCH.

On the other hand, the therapeutic efficacy ADCs could also suffer from drug resistance after multiple treatments due to inherent and acquired resistance or tumor heterogenicity.^[12]^ Although the exact mechanism of drug resistance is still under much investigation, current available clinical data indicate that malignant cells, which are resistant to a particular drug, could still respond to other drugs.^[19]^ Therefore, the attachment of two drugs with different modes of action to an antibody can afford more complex ADCs. This could be co‐delivered to cancer cells to overcome drug resistance,which represents an emerging field in targeted cancer therapy.^[18]^ For example, Levengood and co‐workers have reported a dual‐conjugation strategy for the preparation of novel ADCs including two complementary drugs.^[27]^ In their work, two different auristatin molecules, monomethyl auristatin E(MMAE) and monomethyl auristatin F (MMAF), were selected to conjugate to the cAC10 antibody due to their complementary physicochemical properties and anticancer activities (Figure 14 a,b).^[27]^ Specifically, MMAE is cell‐permeable and exhibits bystander activity capable of killing neighboring antigen‐negative cells.^[133]^ However, MMAE is a substrate for multidrug efflux pump exporters, hence showing diminished activity on cells with high pump expression.^[134]^ In contrast, MMAF is susceptible to drug export, but not cell‐permeable and exhibits negligible bystander effect.^[133]^ In vitro and in vivo experiments demonstrated that the dual‐auristatin ADCs were active on cells and tumors that were refractory to treatment with either of the individual component drugs (Figure 14 c,d). This work highlights the potential of dual modification of antibody for delivering two synergistic and complementary drug payloads for improved antitumor activities, which represents a notable advancement of the ADCs technology.

**Figure 15:**
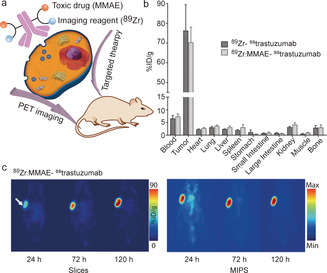
(a) Incorporation of a toxic drug (MMAE) and imaging reagent (^89^Zr) for targeted theranostic applications; (b) Biodistribution data of athymic nude mice bearing HER2‐expressing BT474 breast cancer xenografts after 120 h of administration of the corresponding bioconjugate; (c) PET images of athymic nude mice bearing HER2‐expressing BT474 breast cancer xenografts after the injection of ^89^Zr‐trastuzumab‐MMAE bioconjugate. (b, c) Adapted with permission from ref. [26]. Copyright (2018) American Chemical Society.

Furthermore, dual modification of antibodies also provides great opportunities for simultaneous therapy and diagnostic applications, so‐called theranostics, in which the antibody is conjugated to an anticancer drug and a contrast agent to allow tumor diagnosis and therapy at the same time. In one representative example, Zeglis and co‐workers reported the dual labeling of a HER2‐targeting trastuzumab with a toxin, MMAE, and a positron‐emitting radiometal ^89^Zr for theranostic applications (Figure 15 a).^[26]^ The in vivo experiment indicated that the resulting ^89^Zr‐trastuzumab‐MMAE bioconjugate demonstrated excellent tumor targeting and therapeutic efficacy (Figure 15 b). Importantly, the dual labeled ADCs represents a targeted drug delivery system that could be tracked in vivo using PET providing information of the in vivo biodistribution and real‐time drug doses during the treatment (Figure 15 c).

### 6.3 Dual‐modality imaging

**Figure 16:**
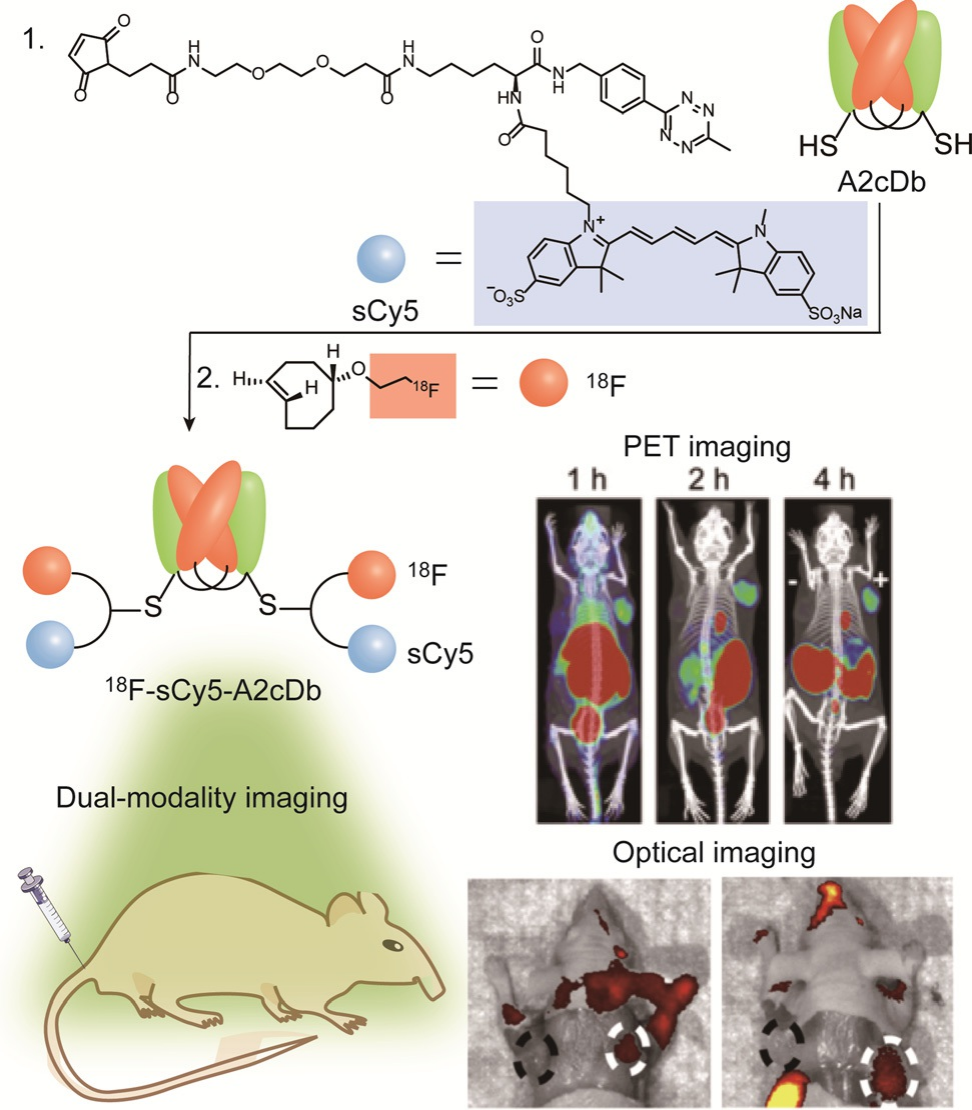
Incorporation of radiometal and ^18^F and sCy5 into A2cDb diabodies for PET imaging and optical imaging. Adapted with permission from ref. [138]. Copyright (2019) Society of Nuclear Medicine and Molecular Imaging.

Molecular imaging is a powerful and invaluable tool for noninvasive visualization of physiological processes that occur in living organisms at cellular levels.^[5]^ Until now, various modern imaging technologies, including optical imaging (OI), magnetic resonance imaging (MRI), positron emission tomography (PET) and computed tomography (CT), have been developed and widely used to monitor the structural, functional and dynamic changes in cancer tissues.^[135]^ Each imaging modality has its own unique strength and intrinsic limitations. Consequently, combining two modal imaging methods has received increasing attention, as it allows the collection of complementary imaging data, thereby improving the reliability and accuracy of the diagnosis. For example, PET can provide real‐time images of tumor lesions as well as monitor their whole‐body distribution and migration, while optical imaging can provide high‐resolution imaging to support surgeons in identifying tumor margins during surgical resection.[^136^, ^137^] By combining both modalities into a single imaging agent, doctors are able to assess the extent of the disease before and after surgery by PET imaging, meanwhile fluorescence imaging can be utilized for image‐guided surgery.^[135]^ Site‐selective dual modification of monoclonal antibodies provides an elegant chemical platform to implement the combination of a radionuclide and a fluorescent dye for dual modality PET and fluorescence imaging. One representative example focused on the conjugation of ^18^F and far‐red dye sulfonate cyanine 5 (sCy5) to anti‐prostate stem cell antigen (PSCA) cysteine mutated diabody A2 via a dual‐modality linker, offering the dual‐modified imaging probe (^18^F‐ sCy5‐A2cDb) (Figure 16).^[138]^ ^18^F‐immuno‐PET showed fast and specific tumor uptake of prostate cancer xenografts, suggesting high‐contrast whole‐body images with organ‐level biodistribution as early as 1 h after injection (Figure 16).^[138]^ Postmortem optical imaging confirmed high‐contrast fluorescence in the PSCA‐expressing tumors and excellent delineation of cancerous cellsfrom surrounding tissue (Figure 16). The dual‐modality imaging provides complemental data, significantly contributing to the reliable and accurate diagnostic applications.

## 7 Conclusion and Outlook

The technical breakthroughs in protein bioconjugation chemistry has served as a major driving force for the elucidation of protein trafficking and interactions in living cells as well as the emergence of protein therapeutics. With the increasing need for personalized treatments to address great challenges in biomedical research such as efficient cell or organ targeting, overcoming drug resistance and reducing systemic toxicity, there is a high demand for protein therapeutics with improved features. Multifunctional protein conjugates provide more than one functionality, and they are expected to enhance in vivo performance of therapeutic proteins by combining different functional groups possessing therapy, diagnostics and imaging properties in a single protein system that is tailored to the requirements of the patient. Nevertheless, dual functionalization of proteins remains much more challenging relative to single functionalization owing to the plethora of reactive functional groups on the protein surface and the requirement for the optimal combination of different orthogonal reactions with high efficiencies. In this review, we have summarized the remarkable progress in both synthetic and genetic engineering strategies that have overcome these significant hurdles and thus allow covalent functionalization of proteins with two different functionalities at distinct sites.

Undoubtedly, dual functionalization of proteins has witnessed significant advancements over the past ten years which offers a new arsenal of functional protein conjugates with advantages over singly‐functionalized proteins in probing protein dynamics, combination therapy and bioimaging. We envision that tremendous efforts will continue to be devoted to enriching the current methodology toolkit by exploring new chemistries to improve the specificity as well as efficiency of dual functionalization or to achieve a higher level of functionalization, for example, triple functionalization. Moreover, the proper combination of different sophisticated strategies, for example, chemical methods, genetic methods, to capitalize the advantage of different approaches from the currently expanding toolbox will provide new insights for the preparation of a myriad of functional nanomaterials. Ultimately, we believe that this will pave the way towards “smart” and “intelligent” protein conjugates that can self‐adapt to the microenvironment of diseases and provide a self‐feedback loop to achieve an output or decision, for example, termination/activation. In this way, current bottlenecks and challenges in therapeutic applications can be addressed with a completely new perspective through rationale chemical design.

## Conflict of interest

The authors declare no conflict of interest.

## Biographical Information

*Lujuan Xu studied chemistry at Zhengzhou University, where she received her Bachelor's (2014) and Master's (2016) degrees. From 2014 to 2016, she conducted her Master's thesis on the synthesis of small organic molecules for optoelectronic devices in the group of Prof. Jian Pei at Peking University. In 2017 she received a CSC scholarship to undertake her PhD with Tanja Weil at the MPIP. Her research focuses on the development of new chemical methodologies for site‐selective modification of proteins*.

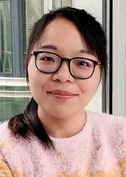

## Biographical Information

*Seah Ling Kuan received her B.Sc (Hons) in 2003 and completed her PhD (Chemistry) at the NUS in 2009. She joined Prof. Tanja Weil in NUS from 2008–2010 as a research fellow. In 2011, she began her independent research as an Alexander von Humboldt fellow in UUlm. She was a group leader in the Institute of Organic Chemistry III at UUlm from 2013–2016. She joined the MPIP in 2016 as a group leader (Protein Therapeutics). Her research focuses on the development of chemical approaches for the innovation of precision protein therapeutics to address devastating diseases such as cancer, inflammation or infections*.

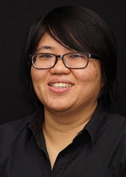

## Biographical Information

*Tanja Weil received her PhD from Mainz University in 2002. She held several leading positions at Merz Pharmaceuticals GmbH (Frankfurt, 2002–2008). She was an Associate Professor at the National University of Singapore (NUS, 2008–2010) and since 2010, director at the Institute of Organic Chemistry III (OC III) at Ulm University (Uulm). In 2016, she was appointed as director of the Department of Synthesis of Macromolecules at the Max Planck Institute for Polymer Research (MPIP) in Mainz, Germany. She has received an ERC Synergy Grant. Her scientific interests focus on innovative synthesis concepts to achieve functional macromolecules, hybrid materials and life‐like systems to solve current challenges in biomedicine and material science*.